# Is a Self-Assessed Questionnaire Useful for the Diagnosis of Ectopic Pregnancy in Hospitalized Patients?

**DOI:** 10.1371/journal.pone.0155054

**Published:** 2016-11-10

**Authors:** Camille Mimoun, Arnaud Fauconnier, Catalina Varas, Cyrille Huchon

**Affiliations:** 1 Department of Gynecology and Obstetrics, Centre Hospitalier Intercommunal de Poissy / Saint-Germain, University of Versailles-Saint-Quentin (UVSQ), Poissy, France; 2 Research unit EA 7285 “Risk and safety in clinical medicine for women and perinatal health”, University of Versailles Saint-Quentin (UVSQ), Poissy, France; VU medisch centrum, NETHERLANDS

## Abstract

**Background:**

Delayed diagnosis of ectopic pregnancy (EP) is responsible for maternal morbidity and mortality. Our objective was to develop and validate decision rules for the diagnosis of EP, in patients in their first trimester of pregnancy with symptoms, based solely on a self-assessment questionnaire.

**Methods:**

From September 2006 to March 2008, 574 patients, who have consulted for acute pelvic pain at the gynecologic emergency department (ED) of five hospitals, completed a Self-Assessment Questionnaire for Gynecological Emergencies (SAQ-GE). We included for our study only women in their first trimester of pregnancy experiencing acute pelvic pain and/or vaginal bleeding who were hospitalized (262 patients). Two-thirds of patients were selected to derive the SAQ-GE EP score which was built on multiple logistic regression. One third of patients were used for internal validation.

**Results:**

Five variables were independently and significantly (p<0.05) associated with EP: no frequent need to change sanitary towels (aDOR = 6.1; 95% CI [2.1–17.8]), duration of bleeding > 24 hours (aDOR = 4,3; 95% CI [1,7–11,0]), pain during coughing (aDOR = 3.1; CI 95% [1,4–6,7]), brown discharge (aDOR = 3.0; 95% CI [1.3–7.1]) and unilateral pelvic pain (aDOR = 2.7; 95% CI [1.3–5.9]). The SAQ-GE ectopic pregnancy score was based on these five criteria with values ranging from 0 to 100. The low-risk group of EP (score<25) had a sensitivity of 95.9% 95% CI [89.8–98,9] and an LR− of 0.2 95% CI [0.1–0.5]. The high-risk group (score>70) had a specificity of 97.4 95% CI [90.9–99.7] and a LR+ of 12.3 95% CI [3.0–49.8]. The percentages of EP observed in the validation sample were: 0% in the low-risk group and 88.9% in the high-risk group.

**Discussion:**

These prediction rules that classify patients in a low-risk or high-risk group may prove useful for triaging pregnant women in their first trimester with symptoms before complementary exams.

## Introduction

In the United States, ectopic pregnancy (EP) is diagnosed in 0.6% to 2.1% of all pregnancies [1] and represents 3% to 4% of all maternal deaths [2]. Among all women in their first trimester of pregnancy attending gynecologic emergency department (ED) for acute pelvic pain and/or vaginal bleeding, 3% are found to have EP, 88.2% intra-uterine pregnancies (IUP) and 8.8% pregnancies of unknown location (PUL) [3].

At present, the diagnosis of EP is made by laparoscopy, which is the gold standard, or by an algorithm combining transvaginal ultrasound (US) and serial serum human chorionic gonadotrophin (hCG) determination [4,5]. In the latter case, transvaginal US and quantitative hCG measurement can be repeated every 48 hours until EP is or is not diagnosed [6]. However, in a crowded ED, hours may elapse between the consultation at the ED and diagnosis of EP by the physician. The risk for patients with a delayed diagnosis of EP is increased morbidity (transfusions, cardiovascular instability, progression of illness) [7].

Triage on the admission to ED by non-clinicians, based only on questioning, could be useful to help identify patients with EP, who should then be given transvaginal US and quantitative hCG measurement as a priority. Clinical prediction models for diagnosing EP have already been developed [8,9] but they cannot be used for triaging patients at ED because they are based not only on the patient’s history but also on physical examination. Furthermore, in these models, history-taking by the physician is liable to be misinterpreted as it is subject to interpretation. Self-assessment questionnaires could avoid this problem. We previously developed a self-assessment questionnaire for gynecological emergencies (SAQ_GE) that describes the characteristics of acute pelvic pain and associated symptoms [10]. It has proved its usefulness for triaging adnexal torsion in patients with acute pelvic pain [11]. The SAQ-GE could be used for triaging EP at the gynecologic ED before consultation with the physician in order to obtain an early diagnosis and prompt treatment.

The aim of this study was to construct a clinical decision rule solely based on the SAQ-GE for identifying patients with a high probability of EP in the gynecologic ED, among pregnant women experiencing early pregnancy symptoms such as acute pelvic pain and/or vaginal bleeding.

## Materials and Methods

The study was approved by the French Department of Higher Education and Research (n°06.336) and by the French National Committee for Information Technology and Individual Liberties (n°906253).

### Self-Assessment Questionnaire for Gynecologic Emergencies

The construction of the SAQ-GE is described in detail elsewhere [10]. It was built using a qualitative method [12] and advice from a panel of French experts. It is an 89-item questionnaire divided into six categories: (i) qualitative description of pain, (ii) intensity of pain, (iii) location of pain, (iv) evolution of pain (v) vaginal bleeding and (vi) associated signs (appendix).

### Derivation and validation of the SAQ-GE ectopic pregnancy score and clinical decision rules

#### Participants

From September 2006 to March 2008, all voluntary patients aged 18 years or more consulting for acute pelvic pain at the participating gynecologic EDs were asked to complete the 89items of the SAQ-GE. The patients were enrolled at five gynecologic EDs in the Paris greater metropolitan region (Poissy-Saint Germain en Laye, Créteil, Port-Royal, Louis Mourier and Versailles). Patients with a history of chronic pelvic pain, no knowledge of French, psychiatric or neurologic diseases were excluded. The ED staff proposed the SAQ-GE to the patients who self-completed it before the initial consultation with the gynecologist and if necessary after appropriate pain management. The nurses then collected the completed questionnaires. They were then anonymized.

For our study we included only pregnant women in their first trimester of pregnancy experiencing acute pelvic pain and/or vaginal bleeding who were admitted to the hospital from the gynecological ED. The pregnancy was diagnosed using a urine pregnancy test for every patient who was not aware of their pregnancy, on arrival at the gynecological ED.

#### Diagnosis of EP

The reference standard for the diagnosis of EP was through laparoscopy. Each patient operated had operative and pathological reports that confirmed the diagnosis.

In the absence of surgery, the diagnosis of EP was made by a non-surgical algorithm [5,6] based on transvaginal US and quantitative hCG measurement. Transvaginal US and hCG measurement were repeated every 48 hours until the definitive diagnosis, IUP or EP, was established.

#### Statistical Methods

The first two-thirds of patients who attended a gynecologic ED were used to derive the clinical decision model (training sample) and the remaining third of patients was used for validation (validation sample).

First, we carried out univariate analysis comparing patients from the training sample with EP and IUP for each variable of the SAQ-GE, using a quantitative (Student's t-test) or qualitative (Chi^2^ test) test as appropriate. We converted variables associated with the presence of EP at a threshold of p<0.10 into dichotomous variables using receiver operating characteristic (ROC) curves. The diagnostic performance of each of these variables (p<0.10) was assessed on the basis of sensitivity (Se), specificity (Sp), positive likelihood ratio (LR+), negative likelihood ratio (LR−) and diagnostic odds ratio (DOR).

Then we used multiple logistic regression analysis to select the best combination of variables that was independently associated with the diagnosis of EP (p<0.05). Variables were selected by a backward, stepwise procedure from those associated with EP in the univariate analysis at a threshold of p<0.10. Adjusted diagnostic odds ratios (aDOR) were calculated with their 95% confidence intervals (95% CI). The stability of each predictive variable in the model was tested using a bootstrap procedure [13]. The performance of the model in the diagnosis of EP was specified by calculating its area under the ROC curve (ROC-AUC).

Next we built a score by rounding up the β coefficients from the multivariate analysis to generate a simple scale [14]. Missing data were considered as absent. The ROC-AUC of the logistic regression model and score model were compared to check that the two values were not significantly different. The predicted probability of EP, Se, Sp, LR+ and LR- were calculated for different threshold values of the SAQ-GE ectopic pregnancy score.

We classified patients as at high risk or low risk of EP by choosing two threshold values of the score to make two classifications, one with sensitivity > 95% and LR+ > 4.0 (rule out) and the other with specificity > 90% and LR− <0.25 (rule in) [8].

Finally, the score was applied to the validation sample. The sensitivity, specificity, LR+ and LR- of the score and the observed probabilities of EP in the high-risk and the low-risk groups were compared with those obtained from the training sample.

Statistical analyses were performed using STATA 13.0 (Stata Corp.; College Station, TX, USA).

## Results

**Fig 1:**
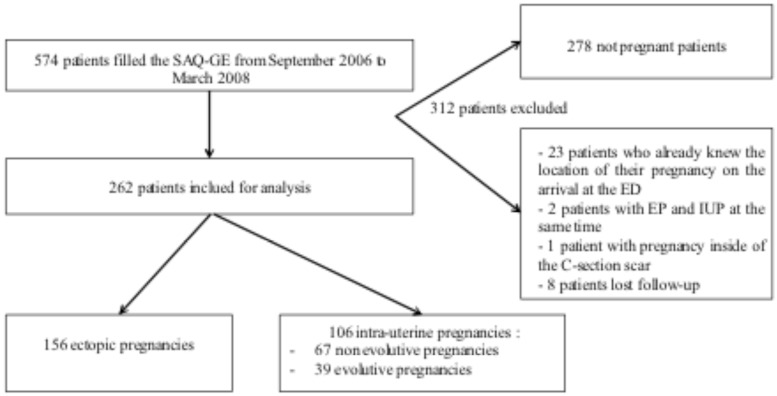
Flow Chart. SAQ-GE, self-assessment questionnaire for gynecological emergencies; EP, ectopic pregnancy; IUP, intra-uterine pregnancy.

During the study period, 574 patients completed the SAQ-GE (S1 Table). Among them, 296 satisfied the inclusion criteria. However, 34 patients were excluded: 23 because we already knew the location of their pregnancy on arrival at the emergency room, 3 because of the exceptional location of their pregnancy and 8 because the location of the pregnancy could not be established (patients lost to follow-up). Consequently a total of 262 patients were included for analysis: 156 patients had an EP and 106 patients had an IUP (Fig 1). The training sample consisted of 174 women, with 97 EP and 77 IUP. The validation sample consisted of 88 women, with 59 EP and 29 IUP. There was no statistical difference between the training and the validation sample concerning the prevalence of EP (p = 0.08), median age, gravidity, parity, history of miscarriage, history of EP and level of pain.

### Derivation

Variables of the SAQ-GE associated with EP in the univariate analysis (p<0.1) were evaluated for their diagnostic performance. Their Se, Sp, LR+, LR- and DOR are shown in Table 1.

**Table 1:** Diagnostic performances of items of the SAQ-GE (p<0,1) associated with EP according to bivariate analysis and multiple logistic regression model for EP in the training sample (N = 174 patients).

	Total, n/N* (%)	Se (%)	Sp (%)	LR+	LR-	DOR [95% CI]	aDOR [95% CI]
Diffuse abdominal pain: No	108/166 (65.05)	71.3	43.1	1.3	0.7	1.88[0.28–1.03]	-
Anal pain: Yes	21/168 (12.50)	18.9	95.9	4.6	0.8	5.45[1.49–20.03]	-
Lumbar pain: No	121/168 (72.02)	77.7	35.1	1.2	0.6	1.89[0.94–3.75]	-
Unilateral pelvic pain: Yes	89/163 (54.60)	67.0	61.1	1.7	0.5	3.2[1.63–6.27]	2.7 (1.3–5.9)
Never experienced such pain before	82/164 (50.00)	59.3	61.6	1.6	0.7	2.34[1.23–4.48]	-
Pain such as uterine contraction: No	89/163 (54.60)	61.8	54.1	1.4	0.7	1.90[1.01–3.59]	-
Pain during palpation of the abdomen: Yes	100/166 (60.24)	66.3	47.3	1.3	0.7	1.77[0.93–3.34]	-
Pain during movement: Yes	104/167 (62.28)	72.0	50.0	1.4	0.6	2.58[1.33–4.99]	-
Pain during coughing: Yes	66/166 (39.76)	49.5	72.6	1.8	0.7	2.59 [1.32–5.09]	3.1 (1.4–6.7)
Sudden onset of pain: Yes	87/165 (52.73)	59.8	56.2	1.4	0.7	1.90[1.01–3.58]	-
Fatigue: No	66/172 (38.37)	44.2	68.8	1.4	0.8	1.75 [0.93–3.31]	-
Vomiting absent or unique	150/172 (87.21)	96.8	24.7	1.3	0.1	10.05[2.64–38.18]	-
Duration of bleeding > 24 hours	61/163 (37.42)	48.9	76.7	2.1	0.7	3.15[1.55–6.40]	4.3 (1.7–11.0)
Bleeding absent or less than periods	121/168 (72.02)	81.5	39.5	1.4	0.5	2.88[1.40–5.92]	-
Frequent need to change sanitary towels: No	132/170 (77.65)	87.4	34.7	1.3	0.4	3.67[1.65–8.17]	6.1 (2.1–17.8)
Evacuation of membranes: No	145/163 (88.96)	95.6	19.4	1.2	0.2	5.25[1.58–17.4]	-
Pain during evacuation of clots: No	151/169 (89.35)	95.7	18.7	1.2	0.2	5.16[1.57–17.04]	-
Evacuation of clots: No	120/170 (70.59)	78.7	39.5	1.3	0.5	2.41[1.21–4.81]	-
Brown discharge: Yes	60/169 (35.50)	48.9	81.3	2.6	0.6	4.18[1.98–8.82]	3.0 (1.3–7.1)

SAQ-GE, self-assessment questionnaire for gynecological emergencies; EP, ectopic pregnancy; Se, sensitivity; Sp, specificity; LR+ positive likelihood ratio; LR- negative likelihood ratio; DOR, diagnostic odds ratio; aDOR, adjusted diagnostic odds ratio after multiple logistic regression, only variables independently and significantly (p<0,05) associated with the diagnosis of EP were retained; 95% CI, 95% confidence interval.

*Because of missing data, total may differ from 174.

**Fig 2:**
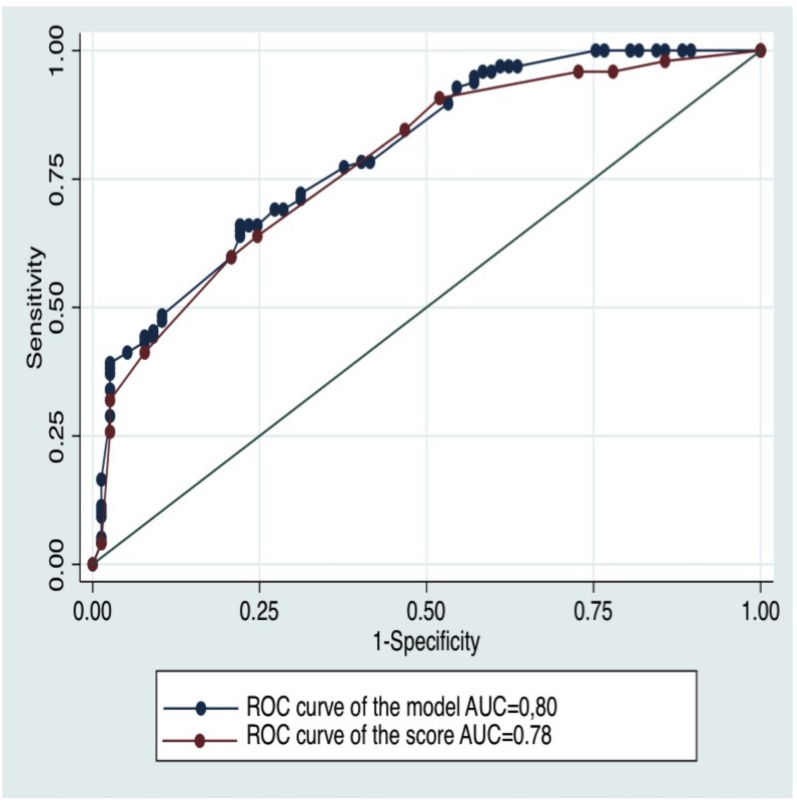
ROC curves of the model and the SAQ-GE EP score in the training sample. ROC, Receiver Operating Characteristic; AUC, Area Under the Curve.

The multiple logistic regression analysis identified five variables independently and significantly (p<0.05) associated with the diagnosis of EP: no frequent need to change sanitary towels (aDOR = 6,1; 95% CI [2.1–17,8]), duration of bleeding > 24 hours (aDOR = 4,3; 95% CI [1,7–11,0]), pain during coughing (aDOR = 3,1; CI 95% [1,4–6,7]), brown discharge (aDOR = 3,0; 95% CI [1,3–7,1]) and unilateral pelvic pain (aDOR = 2.7; 95% CI [1.3–5.9]) (Table 1). All of these variables were stable after 1000 bootstrap replications. The ROC-AUC of this model was 0.80 95% CI [0.74–0.86] (Fig 2).

The SAQ-GE EP score was given by the following equation: Score = (no frequent need to change sanitary towels×30)+(duration of bleeding>24 hours×25)+(pain during coughing×15) +(brown discharge ×15)+(unilateral pelvic pain×15) (Table 2). The ROC-AUC of the score was 0.78 95% CI [0.71–0.85] (Fig 2). There was no significant difference between the ROC-AUC of the score and the ROC-AUC of the model (p = 0.33).

**Table 2:** SAQ-GE EP score.

Variables	Score points
Frequent need to change sanitary towels	
Yes	0
No	30
Duration of bleeding > 24 hours	
No	0
Yes	25
Pain during coughing	
No	0
Yes	15
Brown discharge	
No	0
Yes	15
Unilateral pelvic pain	
No	0
Yed	15
	Score = sum of points/100

SAQ-GE, self-assessment questionnaire for gynecological emergencies; EP, ectopic pregnancy.

We then defined a low and high-risk group of EP (Table 3):

- Patients with a score<25 were defined as a low-risk group. A threshold score value of 25 produced a sensitivity of 95.9% 95% CI [89.8–98.9] and a LR− of 0.2 95% CI [0.1–0.5]
- Patients with a score>70 were defined as a high-risk group. A threshold value of 70 produced a specificity of 97.4 95% CI [90.9–99.7] and a LR+ of 12.3 95% CI [3.0–49.8]

**Table 3:** Diagnostic values of the clinical decision models for the low and high risk groups in the training (N = 174) and validation samples (N = 88).

	Training sample				Validation sample				
	Se (%) [95%CI]	Sp (%) [95%CI]	LR+ [95% CI]	LR- [95% CI]	Se (%)	Sp (%)	LR+	LR-	Probability of EP (%) [95% CI]
Clinical decision model for the low risk group (score < 25)	95.9* [89.8–98.9]	22.1* [13.4–33.0]	1.2* [1.1–1.4]	0.2* [0.1–0.5]	100*	13.8*	1.2*	0*	0
Clinical decision model for the high risk group (score > 70)	32.0 [22.9–42.2]	97.4 [90.9–99.7]	12.3 [3.0–49.8]	0.7 [0.6–0.8]	27.1	93.1	3.9	0.8	88.9 [65.3–98.6]

EP, ectopic pregnancy; Se, sensitivity; Sp, specificity; LR+ positive likelihood ratio; LR- negative likelihood ratio; 95% CI, 95% confidence interval.

* calculated for the absence of EP.

### Validation

The score was calculated for the patients in the validation sample. The low-risk group had a sensitivity of 100% and an LR- of 0. The high-risk group had a specificity of 93.1% and an LR+ of 3.9. The sensitivity, specificity, LR+ and LR- in the validation sample were in the expected range. The observed percentages of EP were: 0% in the low-risk group and 88.9% in the high-risk group (Table 3).

## Discussion

We have developed the first decision rule for triaging patients for EP among pregnant patients hospitalized for acute pelvic pain and/or vaginal bleeding entirely based on a self-assessment questionnaire designed for gynecological emergencies. The clinical model is based on five simple “Yes or No” items, namely: no frequent need to change sanitary towels, duration of bleeding > 24 hours, pain during coughing, brown discharge and unilateral pelvic pain. The clinical decision rule is obtained after combining these items into a score. A score < 25 puts patients in a low-risk group of EP with a sensitivity of 100%, a LR- of 0 and a probability of EP of 0%. A score > 70 puts patients in a high-risk group with a specificity of 93.1%, a LR+ of 3.9 and a probability of EP of 88.9%.

A major strength of our study is the original design of the standardized questionnaire based on self-assessed experience of pain in patients presenting with acute pelvic pain. This differs from using history-taking, which is based on the physician’s perceptions of symptoms and experience. Indeed, the interpretation of history-taking, which necessitates practitioner experience, may be subject to interobserver variability due to cognitive bias [15]. With the SAQ-GE, which was built using the qualitative method of Colaizzi [12], symptoms are collected through questions rigorously formulated by the patients themselves. We can thus have direct access to the symptoms of patients with EP and we can avoid interpretation of the history-taking by the doctor. The SAQ-GE has already been used to build a clinical decision rule for the screening of adnexal torsion and pelvic inflammatory disease in previous studies [11,16]. However, in its actual form, it is too long to fully complete in an emergency unit. We wish to create a simplified version of the SAQ-GE as all of the planned analyses have now been achieved. This questionnaire will contain only the items useful for triage and could be used in routine practice. It could be completed in less time during the admission to EDs by non-clinicians. Indeed, with the new technologies becoming available, alternative methods for filling out the questionnaire are possible, for example using touch pads. The SAQ-GE responses, along with other collected data, would then be instantly processed by a computer and the results used to predict the patients who belong to a specific disease risk group.

Furthermore, the five criteria of our score have good face validity because it was built with common-sense criteria for practitioners involved in gynecological emergencies. They also have good physiopathological plausibility. The pain during coughing, which is a sign of peritoneal irritation, suggests an EP complicated with a hemoperitoneum [9]. The unilateral pelvic pain is a sign of fallopian tube innervation damage by the aortic plexus [17], which is the most common location of EP [18]. The three characteristics of vaginal bleeding reflect bleeding from the fallopian tube.

The major limitation of our study is that we did not include all patients who consulted at a gynecologic ED for acute pelvic pain and/or vaginal bleeding but included only hospitalized patients. This choice was made to limit the potential diagnostic bias consecutive to patients’ loss of follow-up. However, this recruitment strategy may have created a referral bias, in that we included more patients with more severe pathologies. In fact all patients presenting an EP were hospitalized whether they had medical or surgical treatment. On the other hand, patients presenting an IUP were not all systematically hospitalized. Hospitalized patients with IUP may be only those with a more serious condition along with those for whom the initial diagnosis between IUP and EP was difficult (including PUL before definitive diagnosis). This explains the high prevalence of ectopic pregnancy in our study. Differential diagnosis between EP and the more symptomatic IUP is difficult and is of major interest. Even in this selected population, our score is highly discriminant and useful for triaging patients.

Lastly, we performed no cross-validation study with an independent sample. Consequently there is a risk that we overfitted the data when we developed the decision model [19]. To safeguard against overfitting, we used 1000 bootstrap replications to test for instability of the model. The second method we used to avoid overfitting was to divide the population into two, the first two-thirds of patients who went to ED were used to develop the clinical decision model and the remaining third of patients was used to validate it. When an external sample population is not available to validate a model, a sample from the population can indeed be used for validation [20].

Currently, there is no clinical decision rule based solely on questioning for triaging for EP among patients in their first trimester of pregnancy with acute pelvic pain and/or vaginal bleeding. Calculation of the SAQ-GE EP score needs no physical examination nor complementary exams. It could be implemented by non-clinicians for every pregnant patient with acute pelvic pain and/or vaginal bleeding on admission to the gynecologic ED.

The future validation of the simplified version of the SAQ-GE and the decision rule for triaging patients for EP among pregnant patients needs to be carried out in general EDs. The validation of the SAQ-GE EP score needs to be performed on all pregnant patients with acute pelvic pain and/or vaginal bleeding, regardless of whether or not they are hospitalized. After this validation of the score in the general population with future studies, we could even extend its application to general ED, general practitioners or, with the burgeoning new technologies, it could even be used by patients themselves with a host terminal or a touch pad.

All women pregnant in their first trimester of pregnancy experiencing symptoms must have physical examination and complementary exams by a physician. However, depending on the risk groups, the appropriate timing and place to do these exams will be different. Thus, patients classed as at high-risk by the decision rule should see a doctor without delay to have transvaginal US and quantitative HCG measurement in a center open 24 hours a day, seven days a week. On the other hand, when patients are classed as at low-risk complementary exams are still necessary but they are not an emergency.

## Supporting Information

S1 Table Database. (XLS)

## Abbreviations

EP: Ectopic Pregnancy
ED: Emergency Department
SAQ-GE: Self-Assessment Questionnaire for Gynecological Emergencies
IUP: Intra-Uterine Pregnancy (IUP)
PUL: Pregnancy of Unknown Location
US: Ultrasound
hCG: human Chorionic Gonadotrophin
ROC: Receiver Operating Characteristic
AUC: Area Under the ROC Curve
Se: Sensitivity
Sp: specificity
LR+: positive Likelihood Ratio
LR-: negative Likelihood Ratio
OR: Odd ratio
DOR: Diagnostic Odd Ratio
aDOR: Diagnostic adjusted Odd Ratio
CI: Confidence Interval

## References

[1] Hoover KW, Tao G, Kent CK. Trends in the diagnosis and treatment of ectopic pregnancy in the United States. Obstet Gynecol. 2010 3; 115(3): 495–502. 10.1097/AOG.0b013e3181d0c328 20177279

[2] Berg CJ, Callaghan WM, Henderson Z, Syverson C. Pregnancy-Related Mortality in the United States, 1998 to 2005. Obstet Gynecol. 2011 5; 117(5): 1230. 10.1097/AOG.0b013e31821769ed 21705929

[3] Condous G, Okaro E, Khalid A, Lu C, Huffel SV, Timmerman D, et al The accuracy of transvaginal ultrasonography for the diagnosis of ectopic pregnancy prior to surgery. Hum. Reprod. 5 janv 2005; 20(5): 1404–1409. 10.1093/humrep/deh770 15695311

[4] Ankum WM, Van der Veen F, Hamerlynck JV, Lammes FB. Laparoscopy: a dispensable tool in the diagnosis of ectopic pregnancy? Hum Reprod, 1993 8(8): p. 1301–6. 8408532 10.1093/oxfordjournals.humrep.a138246

[5] Mol BW, Hajenius PJ, Engelsbel S, Ankum WM, Van der Veen F, Hemrika DJ, et al Serum human chorionic gonadotropin measurement in the diagnosis of ectopic pregnancy when transvaginal sonography is inconclusive. Fertil Steril. 1998 11;70(5): 972–81. 9806587 10.1016/s0015-0282(98)00278-7

[6] Ardaens Y, Guerin B, Perrot N, Legoeff F. Contribution of ultrasonography in the diagnosis of ectopic pregnancy. J Gynecol Obstet Biol Reprod (Paris). 2003 11; 32(7 Suppl): S28–38. 14699317

[7] Abbott J, Emmans LS, Lowenstein SR. Ectopic pregnancy: ten common pitfalls in diagnosis". The American journal of emergency medicine 1990; 8: 515–5228. 2222596 10.1016/0735-6757(90)90154-r

[8] Buckley RG, King KJ, Disney JD, Ambroz PK, Gormal JD, Klausen JH. Derivation of a clinical prediction model for the emergency department diagnosis of ectopic pregnancy. Acad Emerg Med, 1998 5(10): p. 951–60. 9862584 10.1111/j.1553-2712.1998.tb02770.x

[9] Dart RG, Kaplan B, Varaklis K. Predictive value of history and physical examination in patients with suspected ectopic pregnancy. Ann Emerg Med, 1999 33(3): p. 283–90. 10036342 10.1016/s0196-0644(99)70364-1

[10] Huchon C, Panel P, Kayem G, Bassot A, Nguyen T, Falissard B, et al Is a standardized questionnaire useful for tubal rupture screening in patients with ectopic pregnancy? Acad Emerg Med. 2012 1; 19(1): 24–30. 10.1111/j.1553-2712.2011.01238.x 22221975

[11] Huchon C, Panel P, Kayem G, Schmitz T, Nguyen T, Fauconnier A. Does this woman have adnexal torsion? Hum Reprod. 2012 8;27(8): 2359–64. 10.1093/humrep/des186 22674200

[12] Colaizzi P. Psychological research as the phenomenologist views it Existential phenomenological alternatives for psychology. Oxford University Press, NY; 1978.

[13] Efron B, Gong G. A leisurely look at the bootstrap, the jacknife and cross validation. American Statistician, 1983 37:36–48.

[14] Coste J, Bouyer J, Job-Spira N. Construction of composite scales for risk assessment in epidemiology: an application to ectopic pregnancy. Am J Epidemiol 1997; 145: 278–289. 9012601 10.1093/oxfordjournals.aje.a009101

[15] Fauconnier A, Staraci S, Huchon C, Roman H, Panel P, Descamps P. Comparison of patient- and physician-based descriptions of symptoms of endometriosis: A qualitative study. Hum Reprod 2013; 28:2686Y2694. 23900205 10.1093/humrep/det310

[16] Bouquier J, Huchon C, Panel P, Fauconnier A. A self-assessed questionnaire can help in the diagnosis of pelvic inflammatory disease. Sex Transm Dis. 2014 9;41(9):525–31. 10.1097/OLQ.0000000000000169 25118964

[17] Demco L. Mapping the source and character of pain due to endometriosis by patient-assisted laparoscopy. J Am Assoc Gynecol Laparosc 1998; 5: 241–5 9668144 10.1016/s1074-3804(98)80026-1

[18] Bouyer J, Coste J, Fernandez H, Pouly JL, Job-Spira N. Sites of ectopic pregnancy: a 10 year population-based study of 1800 cases. Hum Reprod 2002; 17: 3224–323. 12456628 10.1093/humrep/17.12.3224

[19] Altman DG, Royston P. What do we mean by validating a prog- nostic model? Stat Med 2000; 19:453Y473. 10694730 10.1002/(sici)1097-0258(20000229)19:4<453::aid-sim350>3.0.co;2-5

[20] Harrell FE Jr, Lee KL, Califf RM, Pryor DB, Rosati RA. Regression modelling strategies for improved prognostic prediction. Stat Med 1984; 3: 143Y152. 6463451 10.1002/sim.4780030207

